# Sensory Re-weighting for Postural Control in Parkinson’s Disease

**DOI:** 10.3389/fnhum.2019.00126

**Published:** 2019-04-17

**Authors:** Kelly J. Feller, Robert J. Peterka, Fay B. Horak

**Affiliations:** ^1^Department of Biomedical Engineering, Oregon Health & Science University, Portland, OR, United States; ^2^Department of Neurology, Oregon Health & Science University, Portland, OR, United States; ^3^Veterans Adminstration Portland Health Care System, Portland, OR, United States

**Keywords:** basal ganglia, sensory integration, feedback, computational model, balance, Parkinson’s disease

## Abstract

Postural instability in Parkinson’s disease (PD) is characterized by impaired postural responses to transient perturbations, increased postural sway in stance and difficulty transitioning between tasks. In addition, some studies suggest that loss of dopamine in the basal ganglia due to PD results in difficulty in using proprioceptive information for motor control. Here, we quantify the ability of subjects with PD and age-matched control subjects to use and re-weight sensory information for postural control during steady-state conditions of continuous rotations of the stance surface or visual surround. We measure the postural sway of subjects in response to a pseudorandom, surface-tilt stimulus with eyes closed, and in response to a pseudorandom, visual-tilt stimulus. We use a feedback control model of the postural control system to interpret our results, focusing on sensory weighting as a function of stimulus amplitude. We find that subjects with PD can re-weight their dependence upon sensory information in response to changes in surface- or visual-stimulus amplitude. Specifically, subjects with PD behaved like age-matched control subjects by decreasing proprioceptive contribution to stance control with increasing surface-tilt amplitude and decreasing visual contribution with increasing visual-tilt amplitude. However, subjects with PD do not decrease their reliance on proprioception as much as age-matched controls for small increases in surface-stimulus amplitudes. Levodopa medication did not affect sensory re-weighting behaviors for postural control. The impairment in PD subject’s ability to respond differently to small changes in surface rotation amplitudes is consistent with an increased threshold for perceiving proprioceptive signals, which may result from decreased signal-to-noise in the dopaminergic pathways associated with sensory processing and/or sensory integration.

## Introduction

Evidence suggests that the basal ganglia are involved in processing and integrating sensory information (Abbruzzese and Berardelli, 2003; Nagy et al., 2006). There is increasing evidence that basal ganglia-related diseases, such as Parkinson’s disease (PD), are associated with kinesthetic deficits, including reduced tactile discrimination, poor joint kinesthesia, asymmetrical spatial pointing, and over-estimating of reaching and stepping when vision is not available (Maschke et al., 2003; Jacobs and Horak, 2006; Tagliabue et al., 2009; Wright et al., 2010). PD also results in motor signs of postural instability, rigidity, tremor and bradykinesia (Horak et al., 1992; Bloem et al., 2001), due to loss of dopaminergic and other neurons throughout the central nervous system, with the severity of motor symptoms related to the amount of nigral-striatal dopamine (Agid, 1991). Although rigidity, tremor and bradykinesia are improved with dopamine replacement therapy, postural control and risk of falls does not improve and may even worsen with levodopa (Horak et al., 1992, 1996).

People with PD fall five times more than age-matched controls (Fasano et al., 2017). Evidence for abnormal postural control in patients with PD comes from studies of unperturbed, quiet stance and studies where balance was perturbed by various sensory stimuli. For quiet stance studies, the effect of PD on postural sway, as quantified by center of pressure (CoP) or center of mass (CoM) displacement, is controversial and may depend upon the sensory conditions and on how CoP or CoM displacement is quantified (Mancini et al., 2011; Curtze et al., 2015; Ozinga et al., 2017; Cruz et al., 2018). Sway area in subjects with PD, when standing with eyes open or closed, can be similar to sway in age-matched controls (Bronstein et al., 1990; Chong et al., 1999a; Bronte-Stewart et al., 2002), especially during early stages of PD (Frenklach et al., 2009). However, sway velocity and jerk have been shown to be increased, even in early PD without medication (Mancini and Horak, 2010; Mancini et al., 2011). In addition, CoP displacement is increased in patients with PD off medication as compared to controls, especially in the mediolateral direction, and levodopa replacement increases CoP displacement (Rocchi et al., 2002). As PD progresses, postural sway area tends to be correlated with the severity of PD (Frenklach et al., 2009).

Studies using perturbed stance have also shown conflicting results. Subjects with PD can generate appropriate sway, even while experiencing a sinusoidal surface displacement (De Nunzio et al., 2007). However, approximately 50% of subjects with PD sway more than age-matched controls with eyes closed on a sway-referenced surface (Bronte-Stewart et al., 2002). Increased sway in subjects with PD under this condition could indicate vestibular dysfunction, because stance with eyes closed on a sway-referenced surface requires increased reliance upon vestibular information. However, peripheral vestibular function is thought to be normal in subjects with PD (Pastor et al., 1993; Bronstein et al., 1996), and a recent study suggests that subjects with PD rely more on vestibular information than control subjects to control postural sway during stance, irrespective of treatment with medication or stimulation of the subthalamic nucleus (Maurer, 2009).

Alternatively, increased sway during sway-referenced conditions with eyes closed could be related to an impaired ability to quickly reorganize the sensory contributions to balance control. To maintain postural stability under suddenly changing sensory conditions, individuals must quickly alter how much they depend upon vision, proprioception, and vestibular information (Peterka and Loughlin, 2004; Jeka et al., 2008; Assländer and Peterka, 2016). It is well known that subjects with PD have a reduced ability to quickly change postural set when sensory or cognitive conditions suddenly change (Chong et al., 2000). For example, subjects with PD take longer than controls to achieve steady-state postural responses following eyes closed to eyes open transitions during sinusoidal surface displacements (Brown et al., 2006; De Nunzio et al., 2007). Furthermore, subjects with PD do not decrease sway with repeated exposure to lateral displacement of visual stimuli (Bronstein et al., 1990). However, many of the studies that manipulate the availability of orientation cues from different sensory systems use short-duration tests. As a result, the observed behavioral differences between subjects with PD and controls may be due to the reduced ability of subjects with PD to quickly adjust when sensory conditions are altered rather than a fundamental inability of subjects with PD to appropriately use sensory information if they are given enough time to adjust.

Our primary goal was to test the fundamental abilities of subjects with PD to adjust to sensory conditions and to regulate sensory integration for postural control in steady-state conditions. We quantified subjects’ relative reliance on visual, vestibular, and proprioceptive information for postural orientation in response to sensory stimuli. Young, healthy subjects typically rely primarily on proprioceptive cues during eyes closed stance but shift toward decreased reliance on proprioception and increased reliance on vestibular cues when the stance is perturbed by support surface rotations of increasing amplitude (Peterka, 2002). Similarly, when visual cues are perturbed by visual surround rotations of increasing amplitude, subjects decrease their reliance on visual orientation cues (Peterka, 2002).

**Figure 1:**
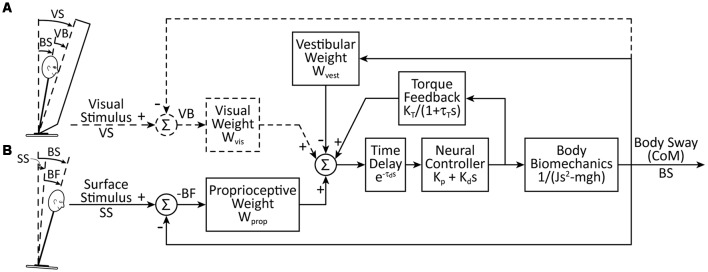
A simplified feedback control model of postural control, including sensory weighting and torque feedback. For the data and analysis we present here, we considered **(A)** a visual-stimulus with a stationary surface and **(B)** a surface-stimulus with eyes closed. The model for the surface-stimulus condition (W_vis_ = 0) is indicated by the solid lines in the schematic. For the visual-stimulus condition, the dashed lines are added to the model and there is no surface stimulus. For the sensory integration component of the model, we constrained W_prop_ + W_vest_ + W_vis_ = 1. The body biomechanics are modeled as an inverted pendulum. The biomechanics, neural controller, torque feedback, and time delay blocks include Laplace transform representations of the differential equations of these model components where s is the Laplace variable.

The quantitative assessment of reliance on a particular sensory modality is made by estimating sensory weighting parameters (Peterka, 2002). The sensory weights are parameters in a linear feedback control system model of the postural control system. Specifically, in our postural control model (Figure 1), the relative reliance on each sensory modality (i.e., vision, proprioceptive, vestibular) is represented as a weighting parameter. Sensory weighting is constrained by W_vis_ + W_vest_ + W_prop_ = 1, where W_vis_ is the visual weight, W_vest_ is the vestibular weight, and W_prop_ is the proprioceptive weight. Therefore, the sensory weight for each of the three weighting parameters can range from 0 to 1. When a subject’s eyes are closed, W_vis_ = 0 and the sensory weighting constraint is reduced to W_vest_ + W_prop_ = 1. Additional parameters of the model include a position- and a velocity-dependent neural control parameter, a neural time delay, and torque feedback implemented as a low-pass filter with a gain and time constant (Figure 1). Previous results showed that young adult subjects with normal sensory function very precisely regulate sensory weights with little variation across subjects and systematically alter these weights as the amplitude of the perturbations change (Peterka, 2002).

Our secondary goal was to determine whether levodopa influences sensory integration. Although dopamine replacement with levodopa medication improves many motor symptoms of PD, its effects on postural control are complex. For example, levodopa improves rigidity, bradykinesia, and tremor, but automatic postural responses (Horak et al., 1996) and postural sway during stance (Nardone and Schieppati, 2006; Rocchi et al., 2006a) worsen with levodopa. Previously, we reported that levodopa does not reduce excessive axial postural tone during stance, despite the reduction of limb rigidity (Wright et al., 2007). However, some components of postural control may improve with levodopa, such as the magnitude of anticipatory postural adjustments prior to movement (Burleigh-Jacobs et al., 1997). There is also evidence that dopaminergic medication further impairs kinesthesia (O’Suilleabhain et al., 2001; Mongeon et al., 2009), although studies have not reported how dopamine replacement affects sensory re-weighting for postural control.

Given that subjects with PD are reported to have various limitations and deficits regarding sensory processing, we hypothesized that sensory re-weighting for postural control would be impaired in subjects with PD. To test this hypothesis, we measured the postural sway of PD and age-matched control subjects while standing in response to pseudorandom surface- and visual-rotations and then used our postural control model to estimate postural control parameters. In this study, we focused primarily on the change of sensory weighting parameters when sensory conditions change. We also tested the additional hypothesis that levodopa medication would improve sensory weighting for postural control.

## Materials and Methods

### Subjects

The Institutional Review Board at Oregon Health and Science University (OHSU) approved the protocol for this experiment, and all subjects gave informed consent prior to participating. Eight subjects with PD (three female) and eight healthy, age-matched controls (two female) were recruited from the Balance Disorders Laboratory database and the Parkinson’s Center of Oregon Clinic at OHSU.

Subjects with PD were selected based on the following inclusion criteria: (1) a diagnosis of idiopathic PD; (2) levodopa responsive, as demonstrated by a lower score on the Unified PD Rating Scale (UPDRS) motor examination when on anti-Parkinsonian medication compared to off medication; and (3) the ability to stand unsupported for 5 min both on and off medication. Subjects with PD were excluded if they had other neurological, sensory, or muscular disorders (e.g., diabetes, peripheral neuropathies, uncorrected visual problems, arthritis, stroke, or seizure).

Control subjects were selected so that no significant differences existed between subjects with PD and controls in age (*p* = 0.79), height (*p* = 0.96), or weight (*p* = 0.71). Additional selection criteria for control subjects were: (1) no known neurological, sensory, or muscular problems; and (2) the ability to stand unsupported for 5 min. Table 1 describes the anthropometric and clinical characteristics of the subjects with PD and the mean anthropometric characteristics of the control subjects. All subjects with PD in the off medication state (PD_Off_), except Subject 1, either had a Hoehn and Yahr score of three or greater or showed impaired balance control in response to a backwards pull on the shoulders [part of the postural instability and gait (PIGD) sub-score of the UPDRS].

**Table 1:** Characteristics of Parkinson’s disease (PD) and control subjects.

										Unified Parkinson’s Disease Rating Scale				
		Age	Height	Weight	DOPA	Years	Most	Hoehn & Yahr	Total Motor	PIGD	Tremor	Bradykinesia	Rigidity	Dyskinesia
Subjects^a^	Sex	(years)	(cm)	(kg)	Equiv^b^.	Since Diag.	affected side	(Off/On)	(Off/On)	(Off/On)	(Off/On)	(Off/On)	(Off/On)	(Off/On)
PD1	F	59	164	83	825	3	R	1/1	^c^/2.5	^c^/1	^c^/0	^c^/1	^c^/0	^c^/0
PD2	M	62	180	105	^d^	5	L	2/1	20.5/4.5	8/4	0/0	2/0.5	2/0	0/0
PD3	M	72	171	71	500	8	R	2.5/2.5	25/16	5/5	9/3	2/2	3/1	0/0
PD4	F	63	163	66	900	13	L	2/1.5	28/11	11/1	1/2	2/0	5/6	0/8
PD5	M	67	174	97	1,180	8	L	3/2	39/21	5/4	6/3	1/1	12/5	0/0
PD6	M	50	182	76	1,000	11	R	3.5/3	49/24	8/5	6/5	3/0	10/3	0/10
PD7	M	70	177	85	1,250	9	L	4/2	59/30	12/6	2/3	3/1	16/6	0/5
PD8	F	69	150	47	1,300	34	R	4/3	63/46	17/11	9/6	4/2	9/8	0/9
Mean PD		64	170	79	994	11		2.8/2	40.5/19.4	9.4/4.6	4.7/2.8	2.4/0.9	8.1/3.6	0/4
Mean Control		64	170	76										

^a^Subjects with PD and controls were matched for age, height, and weight. Biomechanical parameters used in the modeling included J: moment of inertia about the ankle joints and mgh: the product of body mass (not including the feet), the gravity constant, and height of the center of mass (CoM). The mean values for controls and subjects with PD, respectively were J: 76.6 and 79.7 kg m^2^ and mgh: 692 and 717 kg m^2^ s^−2^. Subjects with PD are listed in the order of increasing disease severity, based on the total Unified Parkinson’s Disease Rating Scale (UPDRS) motor score when off medication. ^b^Dopaminergic medications were converted to levodopa equivalents using: DOPA Equiv. (in mg) = levodopa + 0.75*controlled-release levodopa + 100*pramipexole + 16.67*ropinirole + 1.5*amantadine + E_LDE_, where E_LDE_ = 0.25*[levodopa + 0.75*(controlled-release levodopa)] is included if carbidopa/levodopa was coadministered with entacapone and each medication is given in mg of drug administered daily (Nutt et al., 2003). ^c^The UPDRS was not performed for PD1 in the off condition. ^d^A medications list for PD2 was not collected.

We also included data from four younger controls (mean age = 37 years.) that were part of a previously published study (Peterka, 2002).

### Experimental Apparatus

We used a custom-built, balance-testing device for the experiments (Peterka, 2002). The device is comprised of a motor-driven support surface and a motor-driven visual surround. The subject stood on the support surface and faced the visual surround. The support surface can rotate in a toe-up/toe-down direction about the subject’s ankle joints and uses force sensors (Transducer Techniques, Temecula, CA, USA) to measure the subject’s CoP. The visual surround is a half-cylinder, imprinted with a random, complex checkerboard pattern, which can rotate in the anteroposterior direction about the subject’s ankle joint. The balance-testing device measures anterior-posterior body sway by recording the displacement of two sway rods; each sway rod is comprised of a potentiometer (Midori Precisions Co, LTD—Tokyo, Japan) connected to a light metal rod. One sway rod rests in a hook at the subject’s hip height, and the second sway rod rests in a hook at the subject’s shoulder height (see Peterka et al., 2018 for details).

### Experimental Design

Each experiment consisted of six blocks of trials: (1) a calibration trial, (2) surface-stimulus trials, (3) a sway-referenced trial, (4) visual-stimulus trials, (5) a quiet-stance trial, and (6) surface-stimulus trials (repeat). Table 2 lists the blocks of trials and the number of trials comprising each block. The surface-stimulus trials (blocks 2 and 6) and the visual-stimulus trials (block 4) were the focus of the experiment; these blocks tested whether subjects could re-weight sensory information in response to changing sensory stimuli. The sway-referenced trial (block 3) and quiet-stance trial (block 5) tested whether the behavioral characteristics of our subjects with PD were comparable to previously published results.

**Table 2:** Description of trial blocks, conditions, and data analyses.

	Blocks of trials	Trial #	Description of trial conditions			Data analyses
			Subject’s vision	Support surface	Visual surround	
Block 1:	Calibration	1	Eyes Open	Stationary	Stationary	Linear Model
Block 2:	Surface-stimulus trials	2	Eyes Closed	2° PRTS	Stationary	RMS, FRF, Feedback Model
		3	Eyes Closed	1° PRTS	Stationary
		4	Eyes Closed	4° PRTS	Stationary
Block 3:	Sway-referenced trial	5	Eyes Closed	Sway-Referenced	Stationary	Peak-to-Peak CoM Sway
Block 4:	Visual-stimulus trials	6	Eyes Open	Stationary	2° PRTS	RMS, FRF, Feedback Model
		7	Eyes Open	Stationary	1° PRTS
		8	Eyes Open	Stationary	4° PRTS
Block 5:	Quiet-standing trial	9	Eyes Closed	Stationary	Stationary	RMS
Block 6:	Surface-stimulus trials	10	Eyes Closed	2° PRTS	Stationary	RMS, FRF, Feedback Model
	(Reprise of Block 2)	11	Eyes Closed	1° PRTS	Stationary
		12	Eyes Closed	4° PRTS	Stationary

Control subjects performed the experiment once, and subjects with PD performed the experiment twice: once on and once off medication. For off-medication testing, subjects were tested at least 12 h after their last dose of anti-Parkinsonian medication. Five subjects (PD1, PD2, PD3, PD7, and PD8) were tested off medication the first day and on medication the second day. Two subjects were tested on medication the first day and off medication the second day (PD4 and PD6). One subject (PD5) was tested off and then on medication on the same day.

During all testing, except during calibration, subjects wore headphones and listened to an audio book to minimize conscious control of their posture. To prevent injury in the event of losing balance, subjects wore a harness attached to the ceiling of the room, and a researcher spotted them at all times. Subjects rested between trials to minimize fatigue. If a subject fell on a trial, the trial was repeated. If the second attempt also resulted in a fall, a third trial was attempted. All subjects successfully completed all trial types in three attempts, except for the sway-referenced trial. If a subject did not successfully complete a full sway-referenced trial within three attempts, we proceeded to the next block of trials.

#### Calibration Trial

The calibration trial (block 1) defined the relationship between the displacement of the sway rods and the displacement of a subject’s CoM (Peterka, 2002; Peterka et al., 2018). During the 120 s trial, subjects were vocally cued to lean slowly forward and backward through a range of hip and/or ankle angles while they stood with eyes opened on a stationary support surface.

#### Surface-Stimulus Trials

The purpose of the surface-stimulus trials (blocks 2 and 6) was to determine the dynamic characteristics of responses to surface perturbations and to identify how subjects alter their use of proprioceptive orientation cues as a function of stimulus amplitude.

Each surface-stimulus trial was performed with eyes closed. Following 10 s of standing on a stationary surface, the surface rotated according to a stimulus derived from a pseudorandom ternary sequence (PRTS; Davies, 1970). The PRTS was chosen because it has properties similar to white noise (i.e., flat velocity power spectrum over a wide bandwidth), and it appears unpredictable to subjects (see Peterka, 2002 for details). Each trial consisted of four sequential cycles of the PRTS, with each cycle lasting 43.72 s.

In each surface-stimulus block (blocks 2 and 6), subjects performed three surface-stimulus trials with peak-to-peak amplitudes of 2°, 1°, and 4° for the first, second, and third trials, respectively. Subjects rested between trials to prevent fatigue. The surface-stimulus trials of block 6 were a repetition of the surface-stimulus trials of block 2, to determine any learning effect on the subject’s ability to maintain balance.

#### Visual-Stimulus Trials

The purpose of the visual-stimulus trials (block 4) was to determine the dynamic characteristics of responses to visual perturbations and to identify how subjects alter their use of visual cues for spatial orientation as a function of stimulus amplitude.

On each visual-stimulus trial, subjects stood with eyes opened on a stationary support surface looking forward into the visual surround, but not staring at a single point in the pattern. After 10 s, the visual surround rotated according to a PRTS stimulus while the support surface remained stationary. Each trial consisted of four sequential cycles of the PRTS, with each cycle lasting 60.5 s. Subjects performed three visual-stimulus trials with peak-to-peak amplitudes of 2°, 1°, and 4° on the first, second, and third trials, respectively. The subjects rested between trials to prevent fatigue.

A lower bandwidth PRTS was used for the visual stimulus trials than the surface-stimulus trials, because the motor controlling the visual surround had a lower bandwidth than the motor controlling the support surface. Therefore, the PRTS for the visual-stimulus trials contained lower frequencies than the PRTS for the surface-stimulus trials. Consequently, the length of a single cycle of the PRTS was longer for the visual-stimulus trials (60.5 s) than the surface-stimulus trials (43.72 s).

#### Quiet-Standing Trial

The purpose of the quiet-standing trial (block 5) was to quantify the magnitude of the subject’s unperturbed body sway. For the quiet-standing trial, the subject stood upright with eyes closed on the stationary support surface for 120 s.

### Sway-Referenced Trial With Eyes Closed

The sway-referenced trial (block 3) determined whether subjects could change their reliance on sensory information to maintain their balance when vision was absent and relevant proprioceptive cues were suddenly eliminated. In this trial, subjects were required to rely on vestibular information to maintain balance. During the sway-referenced trial, the subjects stood upright on a stationary support surface with their eyes closed. After 60 s, the angular displacement of the lower body, measured using the hip sway rod, was used to control the angular position of the support surface for 60 s; the surface rotated in direct proportion to the subject’s lower body angle with a proportionality constant of 1 (Peterka and Loughlin, 2004). The trial ended with the subject standing quietly on the stationary support surface for another 60 s. These sway-referenced trials are comparable to condition five of the clinical Sensory Organization Test (Horak, 1987; Black et al., 1988).

### Data Analysis

#### Linear Model for Calibration Trial

Using sway data from each subject’s calibration trial, a linear model was used to relate sway rod displacement to CoM displacement, CoM = A_1_S_S_ + A_2_S_H_ + OFF, where S_S_ and S_H_ are body displacements measured using the sway rods at shoulder level and hip level, respectively. The coefficients A_1_ and A_2_ are multipliers for the shoulder and hip displacement, respectively, and OFF is an offset. Based on the assumption that, for slow movements, CoP displacement approximates CoM displacement (Brenière, 1996; Winter et al., 1998), the A_1_, A_2_, and OFF coefficients were determined by minimizing the mean squared error of measured CoP minus the estimated CoM using the fmincon function in MATLAB R2008b (The MathWorks, Inc., Natick, MA, USA). For subsequent trials, the calculated A_1_, A_2_, and OFF coefficients from each subject were used in the linear model, described above, to calculate the CoM displacement from the sway rod measured displacements. A recent publication provides a detailed explanation of this method and includes a Matlab program for the calibration analysis (Supplementary Materials in Peterka et al., 2018).

CoM measurement using this method accounted for the combined motions of the upper and lower body segments and thus provided valid CoM measures whether subjects use ankle or hip strategies. Previous studies have shown that subjects with PD tend to use an inverted pendulum ankle strategy to control standing posture while a mixed hip-ankle strategy is used in control subjects (Baston et al., 2016; Matsuda et al., 2016).

#### Root Mean Square (RMS)

We calculated the root mean square (RMS) for: (1) the CoM displacement for surface- and visual-stimulus trials; (2) the PRTS stimulus displacement for the surface- and visual-stimulus trials; (3) the CoM displacement for successful sway-referenced trials; and (4) the CoP displacement for quiet-standing trials. The signals were zero-meaned prior to calculating their RMS values.

For surface- and visual-stimulus trials, CoM sway data for each subject were first averaged across all available cycles and then an RMS value of the average waveform was calculated. A comparison of the RMS of CoM sway between blocks 2 and 6 indicated that there was no significant learning between blocks in response to surface-stimuli. Therefore, all six cycles from these two blocks were averaged before calculating the RMS, which increased the signal-to-noise ratio of our data. Three cycles of CoM sway data were used for the RMS calculation of responses to visual stimuli for each subject.

#### Analysis for PRTS Trials

Analysis of the surface- and visual-stimulus trials in the time and frequency domains has been previously described (Peterka, 2002). In brief, we considered the subject’s response to the first cycle of the PRTS stimulus to be a transitional cycle during which the subject’s response did not yet reach a steady-state. Therefore, only the second, third, and fourth cycles of a given trial were included in the analysis. For all time domain analyses, we calculated a subject’s average response to each stimulus by averaging the response (CoM displacement) across the three steady-state cycles (cycles 2, 3 and 4). For the frequency domain analysis, we used spectral analyses of each cycle of the stimulus and response in a given trial. The various spectra were averaged across cycles, and further averaged across adjacent frequencies, to yield frequency response function (FRF) and coherence function values at frequency points that were approximately linearly spaced on a logarithmic frequency scale for each stimulus type and for each subject (see Peterka, 2002 for details). FRFs are expressed as gain and phase values that represent the amplitude and timing, respectively, of CoM sway relative to the stimulus across frequency. We computed the mean FRF across subjects and calculated the mean gain and phase curves from the mean FRF.

### Feedback Control Model

#### Model Choice and Optimization

We implemented a model-based interpretation of responses to surface- and visual-stimuli by applying a feedback model of the postural control system, which has been described previously (Peterka, 2003; Cenciarini and Peterka, 2006). Briefly, our model included components for sensory weighting (W_prop_, W_vis_, and W_vest_), neural stiffness (K_p_), neural damping (K_d_), neural time delay (τ_d_), and torque feedback (K_T_) with a low-pass filter time-constant (τ_T_).

Model parameters were estimated using optimized fits to the FRF data. For both the surface- and visual-stimulus trials, fits were made to the three FRFs from each block simultaneously. For the surface-stimulus trials, optimal fits were performed for each subject individually allowing W_prop_ and τ_d_ to vary across the three stimulus amplitudes, but allowing only single values of K_p_, K_d_, τ_T_, and K_T_. These constraints on the optimization fits provided parsimonious descriptions of the experimental FRFs while limiting the total number of free parameters. We also calculated the mean FRF across all subjects and the optimal parameters that fit the mean data.

For the visual-stimulus trials, optimal fits were made only to the mean FRFs for each stimulus amplitude, because the responses to visual-stimulus trials were noisier, and the FRFs fits to individual subjects were not insightful. The visual-stimulus trials were optimized allowing W_vis_ to vary across the three stimulus amplitudes, but allowing only single values of τ_d_, K_p_, K_d_, τ_T_, and K_T_.

### Statistical Analyses

For all statistical analyses, we considered: (1) the effect of PD; and/or (2) the effect of anti-Parkinsonian medication on postural control mechanisms. To determine the effect of PD, we quantified the effect of PD on postural control mechanisms by comparing the behavior of control subjects (C) to PD_Off_. To determine the effect of medication, we quantified the effect of medication on steady-state postural control mechanisms by comparing the behavior of PD_Off_ to subjects with PD on medication (PD_On_). The threshold for significance was *p* < 0.05 for all statistical tests. All statistical analyses were computed in R (The R Project for Statistical Computing; www.r-project.org).

#### Individual Comparisons

Due to the size of our groups, we could not reasonably test whether our data were normally distributed. Therefore, we used the more conservative non-parametric tests to determine significance of individual comparisons. For individual comparisons, the effect of disease (i.e., C compared to PD_Off_) was assessed with the two-sample Wilcoxon rank-sum test, and the effect of medication (i.e., PD_Off_ compared to PD_On_) was assessed with the paired Wilcoxon signed-rank test. For statistical testing of the clinical measures of the UPDRS, and sub-scores of the UPDRS, we used a single-sided distribution, because we were testing whether there was an improvement in the UPDRS score on vs. off medication. All other individual comparison tests were calculated with a two-sided distribution.

#### Repeated-Measures ANOVAs

We used repeated-measures ANOVAs (i.e., using aov in R) to test the hypotheses that PD influences: (1) CoM displacement in response to surface-stimuli, (2) W_prop_ in response to surface-stimuli, and (3) the time-delay parameter in our feedback control model. In the above three ANOVAs, RMS of CoM displacement, W_prop_, and τ_d_ were the dependent variables, respectively. We included factors for group (i.e., Controls vs. PD_Off_), stimulus amplitude (i.e., 1°, 2°, and 4°), and an interaction effect (i.e., group × stimulus amplitude). We also included a random factor for subject, accounting for the fact that each subject performed the experiment for all three stimulus amplitudes.

Similarly, we used a repeated-measures ANOVA to test the hypothesis that levodopa influenced W_prop_ in response to surface-stimuli. This ANOVA included factors for medication conditions (i.e., ON vs. OFF), stimulus amplitude, and an interaction effect. This ANOVA also included a random factor for subject, accounting for the fact that each subject performed the experiment for all three stimulus amplitudes and two medication conditions.

## Results

### Clinical Balance and Quiet Stance Measures

**Figure 2:**
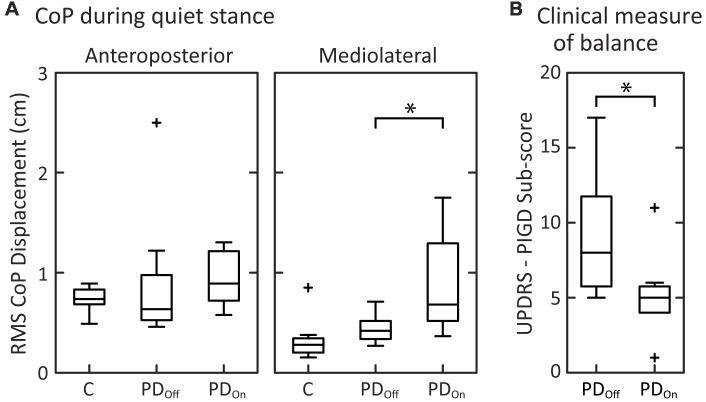
Clinical measures of balance and center of pressure (CoP) measurements. **(A)** Box and whisker plots of the CoP during quiet stance for the anteroposterior and mediolateral directions. **(B)** Box and whisker plots of the postural instability and gait disorders components (PIGD) of the Unified Parkinson’s Disease Rating Scale (UPDRS; *N* = 7; PD1 is not included due to incomplete data for the UPDRS off medication). For each subject group, the center line is the median, the bottom of the box is the 25th percentile (Q1), and the top of the box is the 75th percentile (Q3). The whiskers extend to include all data points that are within the range defined by Q1−1.5(Q3 - Q1) and Q3 + 1.5(Q3 - Q1). Data points that extend beyond the whiskers are defined as outliers and denoted with a “^+^.” Brackets with “*” indicate significant differences between mean values.

Quiet stance measures of sway and clinical signs on and off levodopa were similar to those reported in previous studies. During quiet stance with eyes open, RMS of CoP displacement was not significantly different between PD_Off_ and control subjects in the anteroposterior direction (Figure 2A; *p* = 0.38) or in the mediolateral direction (*p* = 0.083). Levodopa increased the RMS of CoP displacement from PD_Off_ to PD_On_ in the mediolateral (*p* = 0.023), but not in the anteroposterior direction (*p* = 0.20), as shown previously (Mitchell et al., 1995; Rocchi et al., 2002; Curtze et al., 2015).

The peak-to-peak CoM sway of the PD_On_ and elderly control subjects, who did not fall on the sway-referenced trial, were consistent with previously published results (Chong et al., 1999a). Two of the eight controls, three of the eight PD_On_ subjects, and four of the eight PD_Off_ subjects fell on all three attempts of standing on a sway-referenced surface with eyes closed. This result is consistent with previous results (Bronte-Stewart et al., 2002; Frenklach et al., 2009) showing that a subset of patients with PD fall on all sway-referenced attempts, regardless of disease severity.

As expected, levodopa improved the UPDRS III Motor score (*p* = 0.011). Levodopa also improved the PIGD sub-score of the UPDRS (Items 26–30; Figure 2B; *p* = 0.018) as well as rigidity (Item 22; Table 1; *p* = 0.021), and bradykinesia (Item 31; Table 1; *p* = 0.027).

### Stimulus-Evoked Sway

**Figure 3:**
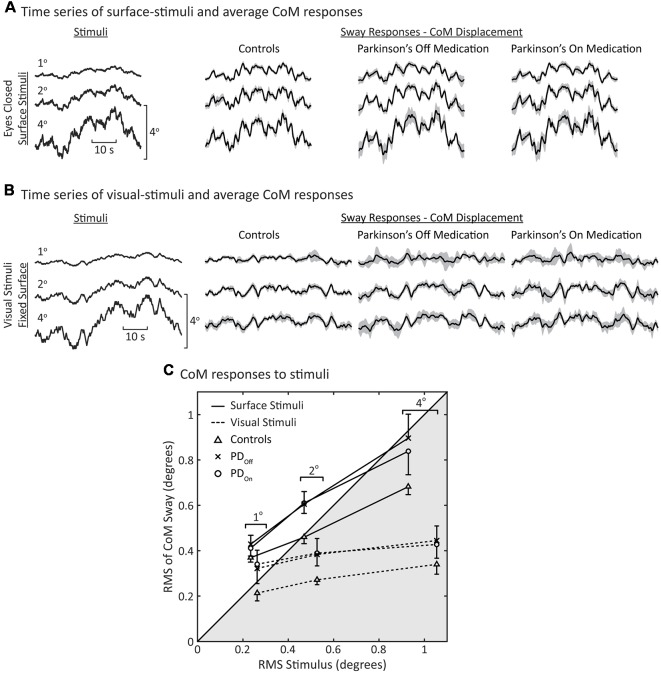
Sway during surface and visual stimuli. Time series of average center of mass (CoM) responses to **(A)** surface stimuli and **(B)** visual stimuli. The shaded gray regions represent the 95% confidence intervals of the mean response across subjects. **(C)** Root mean square (RMS) of the CoM sway vs. RMS of the stimulus. Each data point represents the average behavior across subjects (*N* = 8 for each group). Data in the unshaded half of the figure represents CoM responses that are larger than the stimulus (ratio >1); the shaded portion of the plot represents responses that are smaller than the stimulus (ratio <1). Note that the surface and visual stimuli were different pseudorandom ternary sequences (PRTSs; see Stimuli columns in **(A,B)** and see “Materials and Methods” section), consequently the RMS of the stimuli are slightly different for the same peak-to-peak stimulus amplitude. The error bars denote standard errors for the surface-stimulus data. For clarity, only single-sided error bars are shown.

For all stimulus amplitudes, control subjects and subjects with PD on and off medication tended to orient the angular displacement of their body CoM to either the moving support surface (Figure 3A) or visual surround (Figure 3B). At the lowest stimulus amplitudes, CoM sway was larger than the surface-stimulus amplitude (Figure 3A, row 1). CoM sway increased with increasing surface-stimulus amplitude. However, control subjects did not sway as much as subjects with PD at the largest surface-stimulus amplitude (Figure 3A, row 3). Control subjects swayed less than the surface-stimulus amplitude, and PDs swayed approximately the same as the surface-stimulus amplitude in response to the 4° surface-stimulus. CoM sway was similar between PD_Off_ and PD_On_ for all surface-stimulus amplitudes (Figure 3A, two rightmost columns). The variability across subjects of the CoM sway was larger in PD_Off_ and PD_On_ than control subjects (Figure 3A, gray shaded regions).

As with surface stimuli, all subjects tended to orient their CoM sway to the visual stimulus (Figure 3B). Overall, responses to visual stimuli were smaller in magnitude than responses to surface stimuli (Figures 3A,B). There were no obvious differences in the average sway of PD_Off_, PD_On_, and control subjects in response to visual-stimuli. However, the variability across subjects was greater for subjects with PD than controls in response to visual stimuli (Figure 3B, gray shaded regions).

To quantify the degree by which subjects increased body sway in response to increased surface-stimulus amplitudes, we calculated the RMS of the CoM sway and stimulus (Figure 3C). There was an increase in the RMS of CoM sway with increasing surface-stimulus amplitude for PD_Off_, PD_On_, and control subjects. This increase in the RMS of CoM sway was smaller than the increase in the RMS of the stimulus. PD_Off_, PD_On_ and control subjects had similar sway in response to 1° surface stimuli, and all subject groups swayed more than the stimulus amplitude in response to the 1° surface stimulus (Figure 3C, 1° data in white region).

For larger surface-stimulus amplitudes, subjects with PD showed greater RMS sway than controls (Figure 3C, solid lines). Statistical analysis based on a repeated-measures ANOVA showed a significant main effect of stimulus amplitude (*p* < 0.001) on the RMS of CoM sway. The main effect of disease on the RMS of CoM sway was not significant (*p* = 0.059), nor was the interaction between disease and stimulus amplitude (*p* = 0.11).

For visual stimuli, the RMS sway was similar for PD_Off_ and PD_On_ with controls having lower RMS sway than subjects with PD at all stimulus amplitudes (Figure 3C, dashed lines). For subjects with PD and controls, RMS sway levels showed moderate increases with increasing visual stimulus amplitude and these sway levels were smaller than for surface stimuli.

### Postural Dynamics

#### Individual Responses to Surface-Stimuli

**Figure 4:**
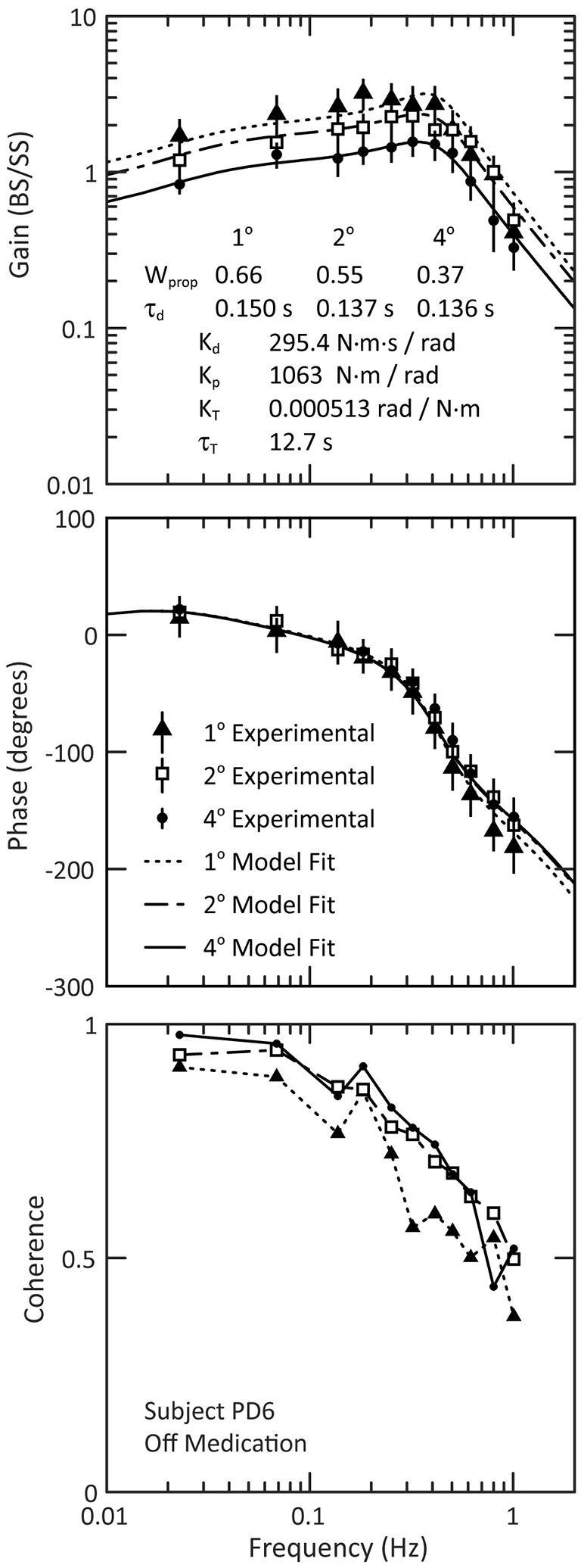
Example of experimental frequency response functions and model fits for PD subject PD6 off medication. The experimental data are shown for the gain (top figure) and phase (middle figure), and the 95% confidence intervals of the mean are indicated by the vertical lines through each data point of the gain and phase curves. The bottom figure shows the coherence of the experimental data. Note that the lines in the coherence plot (bottom figure) are for visualization of the experimental data; these lines are not related to the model fits.

We used a frequency domain analysis to characterize each subjects’ postural sway over a range of perturbation frequencies. Figure 4 shows examples of experimental FRFs and model fits for an individual PD_Off_ subject’s response to three surface-stimulus amplitudes. The main feature of the FRF is a decreasing gain with increasing stimulus amplitude (Figure 4, top). The phase is similar across stimulus amplitude for most frequencies, except at higher frequencies where there is a slightly larger phase lag for the 1° than for 2° and 4° stimulus amplitudes (Figure 4, middle). The coherence of the experimental data decreases with increasing frequency (Figure 4, bottom). Figure 4 also shows the model fits to the experimental data (see Figure 1 for model). The model fits replicate the main features of the experimental data, with decreasing dependence on proprioception (smaller W_prop_) accounting for the gain decrease as stimulus amplitude increases and decreasing time delay (τ_d_) accounting for phase changes at higher frequencies.

#### Group Responses to Surface-Stimuli

**Figure 5:**
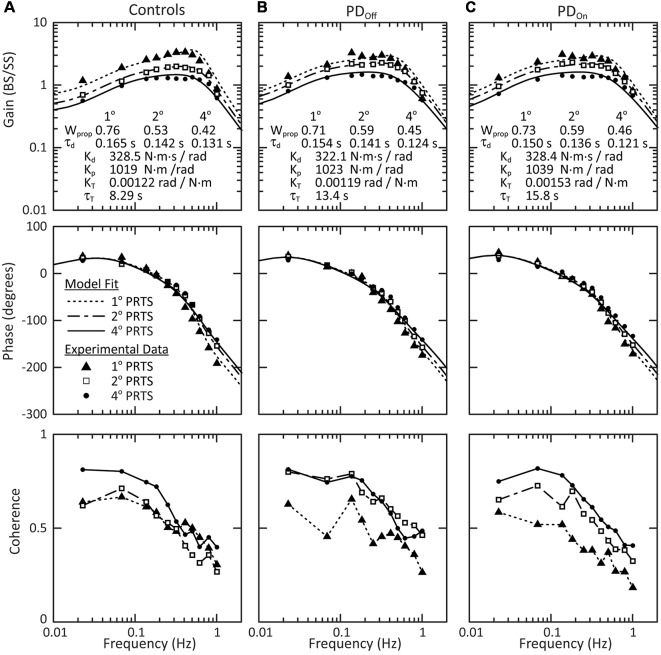
Mean behavior across subjects in response to surface stimuli with eyes closed. Gain, phase and coherence curves for **(A)** control subjects, **(B)** subjects with PD off medication, and **(C)** subjects with PD on medication. The data points denote experimental data for the mean behavior across subjects and the curves on the gain and phase plots are the model fits to this mean behavior. The parameters of the model fits are listed in the gain plots. Data for all three stimulus amplitudes were fit simultaneously, with a W_prop_ and τ_d_ for each stimulus amplitude, and a single K_d_, K_p_, K_T_, and τ_T_ across all stimulus amplitudes. The lines connecting data points in the coherence plots (bottom row) are for visualization and are not related to model fits.

The results observed for the individual PD_Off_ subject (Figure 4) were representative not only of the mean behavior across all PD_Off_ subjects but also of the mean behavior for the control and PD_On_ subjects. The mean behavior of each subject group included a decrease in the gain with increasing stimulus amplitude, and small phase changes at higher stimulus frequencies (Figure 5). There were no qualitative differences between PD_Off_, PD_On_, and control subjects in the gain, phase or coherence curves for any stimulus amplitude. Consequently, the parameters of the model fits to the mean group data were similar for PD_Off_, PD_On_, and control subjects.

#### Model-Based Interpretation for Surface Stimuli

**Figure 6:**
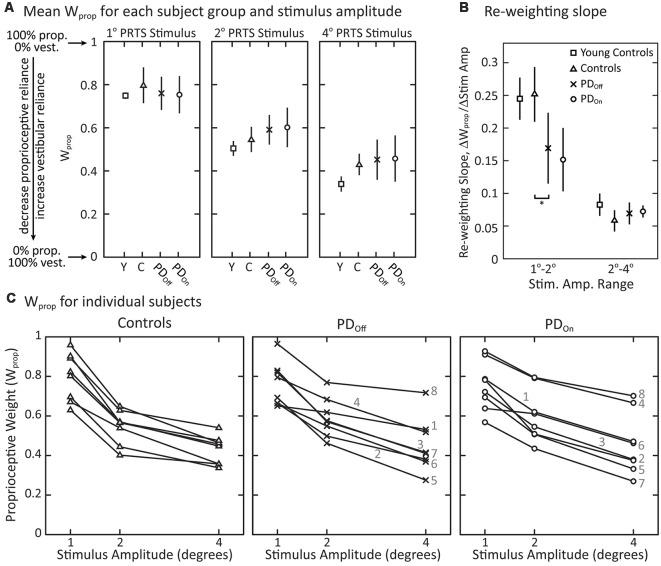
Proprioceptive weighting in response to surface-stimuli with eyes closed. **(A)** Sensory weighting across subject groups and stimulus amplitudes. Data are shown for young controls (Y: *N* = 4; age range 28–47 years), older controls (C: *N* = 8; age range 57–77 years), and subjects with PD off (PD_Off_: *N* = 8) and on (PD_On_: *N* = 8) medication. A proprioceptive weight of 1 indicates 100% reliance on proprioceptive information, while a weight of 0 indicates 100% reliance on vestibular information. The symbols represent mean behavior for each group, and the error bars denote the 95% confidence interval of the mean. **(B)** Slope factor representing the normalized change in proprioceptive weights between 1° and 2° surface-stimuli and between 2° and 4° surface-stimuli for each subject group. All changes in W_prop_ are positive, indicating a decrease in W_prop_ for increasing stimulus amplitude. The error bars denote the 95% confidence intervals of the mean and “*” indicates a significant difference between subjects with PD off medication and age-matched controls. Young controls were not included in statistical tests. **(C)** Sensory weighting in individual subjects in response to surface stimuli for controls, and subjects with PD off medication and on medication. Each data point represents a single subject at the given stimulus amplitude. Lines connect the data points for an individual subject across stimulus amplitudes. For subjects with PD, the numbers 1–8 correspond to the subject identifiers in Table 1 (e.g., PD1 denoted as 1). All subject groups showed significant decreases in W_prop_ with increasing stimulus amplitude.

There were no significant effects of disease (controls vs. PD_Off_) on sensory-to-motor characteristics of posture control: K_p_ (*p* = 0.88), K_d_ (*p* = 0.80), τ_d_ (*p* = 0.74), K_T_ (*p* = 0.64), or τ_T_ (*p* = 0.083). Medication (PD_Off_ vs. PD_On_) also did not significantly affect K_p_ (*p* > 0.99), K_d_ (*p* = 0.38), τ_d_ (*p* = 0.82), K_T_ (*p* = 0.20), or τ_T_ (*p* = 0.25). All subject groups showed a significant decrease in proprioceptive weighting (W_prop_) with increasing surface-stimulus amplitude (Figure 6A; *p* < 0.001). Although the mean W_prop_ was larger for controls than for subjects with PD at 1° and smaller for controls than for subjects with PD at 2° and 4°, there was no significant main effect of disease (controls vs. PD_Off_) on W_prop_ (Figure 6A; *p* = 0.84), and there was no interaction effect between group and stimulus amplitude (*p* = 0.28). In addition, there was no main effect of medication on W_prop_ (*p* = 0.92) and no interaction between medication and stimulus amplitude (*p* = 0.82).

Viewing the changing W_prop_ values of individual subjects across stimulus amplitude revealed some qualitative differences between subjects with PD and age-matched control subjects (Figure 6C). There was more inter-subject variability in W_prop_ in subjects with PD than in control subjects in response to the 4° surface-stimulus amplitude (Figure 6C). This inter-subject variability in the PD groups was due, in part, to the large proprioceptive weights for a single PD_Off_ subject (PD8) and two PD_On_ subjects (PD4, PD8) across all stimulus amplitudes. In fact, when off medication, PD8 weighted proprioception more than any control subject for the 2° and 4° surface-stimulus amplitudes. In the on medication condition, PD4 and PD8 had a larger W_prop_ than any controls for the 2° and 4° surface-stimuli. Medication noticeably changed W_prop_ in two of the eight subjects with PD (Figure 6C); W_prop_ increased in PD4 and decreased in PD7 across all stimulus amplitudes when on vs. off medication. Thus, large changes in W_prop_ occurred with medication in some individual subjects, but the direction of change was not systematic. In addition, three (PD1, PD4, PD8) of the four PD_Off_ subjects who fell on all attempts at sway-referenced trials had larger W_prop_ in the 2° and 4° surface-stimulus trials than the PD_Off_ subjects that did not fall. One of the three PD_On_ subjects (PD8) who fell on all attempts at sway-referenced trials also had larger proprioceptive weights for all surface-stimulus trials than PD_On_ subjects who did not fall.

Additional differences between control and individual subjects with PD were related to how well they changed proprioceptive weighting between surface-stimulus amplitudes (Figure 6C). To quantify this difference in W_prop_ between control and subjects with PD, we computed the slope of the proprioceptive weights between successive surface-stimulus amplitudes. PD_Off_ subjects’ slope of proprioceptive weighting was less than controls between 1° and 2° surface-stimulus amplitudes (Figure 6B; *p* = 0.038). However, there was no difference between PD_Off_ and control subjects in the slope of proprioceptive weighting between 2° and 4° surface-stimulus amplitudes (*p* = 0.51).

#### Group Response to Visual-Stimuli

**Figure 7:**
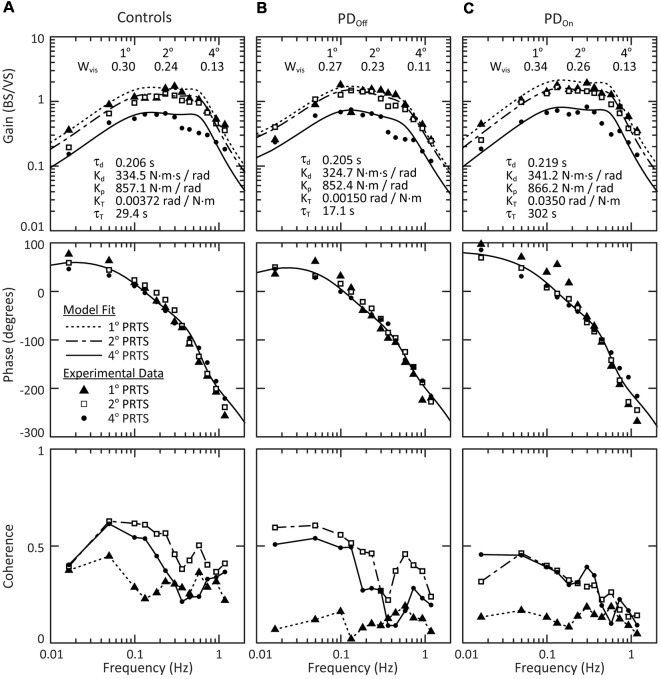
Mean behavior across subjects in response to visual stimuli. Gain, phase and coherence plots for **(A)** control subjects, **(B)** subjects with PD off medication, and **(C)** subjects with PD on medication. The data points denote experimental data for the mean behavior across subject and the curves on the gain and phase plots are the model fits to this mean behavior. The parameters of the model fits are listed on the gain plots. Data for all three stimulus amplitudes were fit simultaneously, with a W_vis_ for each stimulus amplitude and a single τ_d_, K_d_, K_p_, K_T_, and τ_T_ across all stimulus amplitudes. The lines connecting data points on the coherence plots (bottom row) are for visualization and are not related to the model fits.

In response to visual-stimuli, both PD and age-matched control subjects had decreasing FRF gains with increasing stimulus amplitude (Figure 7, top row). For a given stimulus amplitude, the gain in response to a visual stimulus was lower than the gain in response to a surface stimulus (compare Figures 5, Figure 7). In contrast to responses to surface stimuli, the phase curves in response to visual stimuli were similar across stimulus amplitude for all frequencies (Figure 7, middle row). Individual subject’s responses to visual stimuli were small and variable. Consistent with this low gain and high variability, the coherence of the experimental data was consistently low for individual subjects (not shown) and the mean coherence across subjects (Figure 7, bottom row). Therefore, we did not consider statistics for individual subject responses to visual stimuli, and conclusions were based on a qualitative assessment of the average response across subjects.

**Figure 8:**
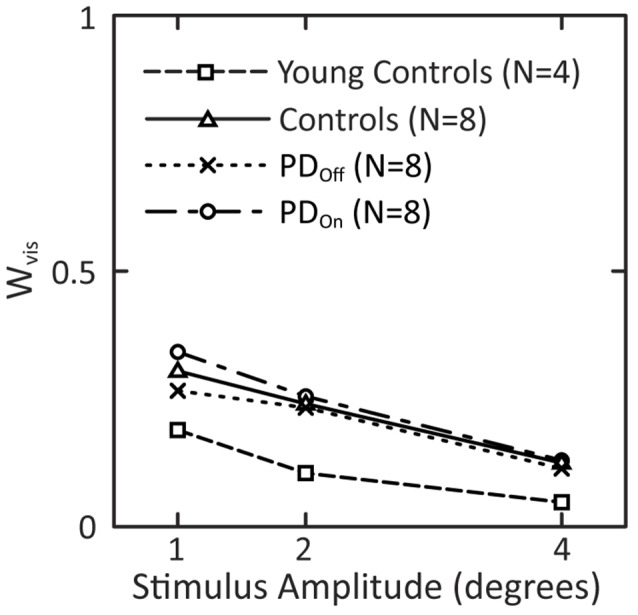
Visual weights in response to visual-stimuli of different stimulus amplitudes. Data for subjects with PD and age-matched controls are taken from the model fits to the mean FRF data in Figure 7. Visual weights from fits to mean FRF data from four young controls are added to this plot.

All groups showed a mean tendency to decrease reliance on visual information with increasing visual-stimulus amplitude, as indicated by a monotonic decrease in W_vis_ with increasing visual-stimulus amplitude (Figure 8). Neither PD nor levodopa influenced visual weighting in response to visual stimuli (Figure 8).

#### Effect of Aging on Sensory Weighting

There was a tendency for our age-matched control subjects to have larger mean proprioceptive weights than younger controls (data from Peterka, 2002) in response to each surface stimulus (Figure 6A). On average across surface-stimulus amplitudes, W_prop_ for older controls was 1.1 times the W_prop_ for younger controls. The difference in the mean W_prop_ between older and younger control subjects was greatest for the 4° surface-stimulus amplitude. In addition, subjects with PD had larger proprioceptive weights than younger subjects for the 2° and 4° stimulus amplitudes (Figure 6A). The difference in proprioceptive weighting between 1° and 2° surface stimuli were similar for age-matched and younger control subjects, and both control groups had noticeably larger slopes than subjects with PD (Figure 6B).

The mean visual weight, W_vis_, at each individual amplitude was smaller for young control subjects than either PD or age-matched control subjects (Figure 8). W_vis_ for older controls was on average 2.2 times larger than the W_vis_ for the younger controls. In addition, W_vis_ for PD_On_ and PD_Off_ subjects was on average 2.0 and 2.3 times, respectively, larger than W_vis_ for younger controls.

## Discussion

Despite profound balance and motor control deficits, our results demonstrate that subjects with PD can re-weight proprioceptive, visual, and vestibular information for postural control when sensory conditions change. Each PD and age-matched control subject decreased reliance on proprioceptive information as the surface-stimulus amplitude increased (Figures 6A,C). However, subjects with PD did not change proprioceptive weights as much as control subjects between the smallest surface-stimulus amplitudes (Figure 6B).

To appreciate the functional significance of the relatively small differences in sensory weighting between subjects with PD and controls, comparisons can be made to sensory weight changes associated with other neurological deficits. Specifically, sensory weights have been measured in subjects with bilateral (Figure 10 in Peterka, 2002) and unilateral vestibular loss (Figure 4 in Peterka et al., 2011) using similar methods. Bilateral vestibular loss subjects are 100% reliant on proprioception (W_prop_ = 1) and are unable to change weight when amplitudes of surface stimuli change on tests performed with eyes closed. Bilateral vestibular loss subjects were also unable to change visual weights with changing visual stimulus amplitude. Unilateral vestibular loss subjects were able to decrease W_prop_ with increasing surface amplitude, but their W_prop_ values were larger than age-matched controls by an average value of 0.28. Additionally, there was very little overlap in the distributons of W_prop_ values from unilateral loss and control subjects. These results in vestibular deficient subjects are in contrast to the substantial overlap of W_prop_ values in subjects with PD and controls at all stimulus amplitudes (Figure 6C) and emphasize the minimal effect of PD on sensory weighting and re-weighting.

The subjects in our study would be considered at increased risk for falls compared to age-matched controls given that they had moderate to severe PD (UPDRS Motor score 20–63), were diagnosed from 3 to 36 years ago, were dependent upon levodopa, and seven out of eight showed clinically apparent balance and gait problems. The moderately-to-severely affected patients in our study showed larger spontaneous sway in the mediolateral (ML) direction, especially when taking their levodopa medication, consistent with previous studies (Rocchi et al., 2002) and possibly related to dyskinesia (Chung et al., 2010; Curtze et al., 2015). In addition, fewer of our subjects with PD than age-matched controls were able to stand unsupported on a sway-referenced surface with eyes closed, especially when off levodopa, which is similar to other studies (Bronte-Stewart et al., 2002).

Other studies have shown that subjects with PD as severe as those in our study, have significant impairments in automatic postural responses, postural instability during gait, and in anticipatory postural adjustments (Horak et al., 1996; Rocchi et al., 2006b; Tagliabue et al., 2009). Despite including subjects with PD with a range of disease severity, we did not see any significant correlations between disease severity and impairments in sensory weighting. However, there was some indication that disease severity may be related to sensory weighting in that the two subjects with PD (PD4 and PD8) who had the disease the longest and had among the worst clinical PIGD scores in the UPDRS (Table 1) also relied more on proprioception than the other subjects with PD when on or off medication (Figure 6C). Future studies with larger sample sizes would be necessary to investigate this possible relatationship of severity and sensory weighting with other relevant outcome measures such as incidence of falls.

### Sensory Integration and Sensory Transitions

Our results demonstrate that subjects with PD are capable of re-weighting sensory information for postural control. This is consistent with a previous study concluding that subjects with PD can integrate sensory information to successfully perform a turning task before and after walking on a circular treadmill (Earhart et al., 2007). However, previous findings from our laboratory show that subjects with PD take more trials than control subjects to switch postural synergies when sensory conditions change (Horak et al., 1992; Chong et al., 1999b,c, 2000). For example, subjects with PD do not immediately inhibit ankle muscles when holding a handle or sitting on a stool during surface perturbations (Schieppati and Nardone, 1991; Horak et al., 1992, 1996). In addition, on the first trial of each of the sensory organization tests, subjects with PD fall more often than controls (Chong et al., 1999a). However, subjects with PD improve with repeated exposure to each sensory condition, such that by the third trial of a particular sensory condition, subjects with PD reach near control levels (Chong et al., 1999a). The third trial of each sensory condition is most similar to the steady-state conditions of our experiment. Therefore, this previous result is consistent with the mostly appropriate steady-state performance of our subjects with PD when compared to age-matched controls.

In response to changing surface-stimulus amplitudes in the absence of vision, our subjects with PD demonstrated an ability to re-weight proprioceptive information in a similar manner as age-matched controls. This result indicates that vision is not required for subjects with PD to generate appropriate steady-state postural responses. On the contrary, previous studies suggest that subjects with PD are more visually-dependent than age-matched controls, especially when visual information is misleading (Bronstein et al., 1990). For example, subjects with PD consistently undershoot voluntary arm movements and involuntary postural stepping responses when they cannot see their limbs (Jacobs and Horak, 2006; Tagliabue et al., 2009). It has also been shown that body sway is more driven by sinusoidally moving visual surrounds in subjects with PD than age-matched controls (Maurer et al., 2003). Although our subjects with PD showed slightly greater sway than our age-matched controls (Figure 3C), both groups showed sway increases with increasing visual-stimulus amplitude. These results differ qualitatively from the Maurer study in that their control subjects showed essentially no increase in sway with increasing visual-stimulus amplitude and showed significantly less sway than their subjects with PD. The difference between our results and the Maurer study may be due to the unusually young ages of their subjects with PD and hence the ages of their age-matched controls (48 years mean age of both groups). Consistent with age accounting for increased sensitivity to visual stimuli are the results in Figure 8 showing that W_vis_ measures for young controls are about half the value of W_vis_ for our age-matched controls. That is, normal aging may result in a shift toward increased reliance on vision for balance; early onset PD may accelerate the shift, but older subjects show a similar reliance on vision independent of disease state.

Furthermore, some of our subjects with PD performed poorly on sway-referenced surface trials with eyes closed. In these trials, subjects must rely on vestibular information, as vision is absent and the sway-referenced surface minimizes proprioceptive cues. However, vestibular function has been shown to be normal in PD (Pastor et al., 1993). In fact, it has been shown that subjects with PD with deep brain stimulation weight proprioceptive information less and over-weight vestibular sense for postural control compared to age-matched controls (Maurer, 2009). Thus, it is unlikely that either vestibular dysfunction or an inability to use vestibular information for postural control account for the poor performance in subjects with PD on eyes closed sway-referenced trials. Consistent with our sensory re-weighting results, we suggest that most subjects with PD have normal vestibular function and are able to utilize it if given enough time to adjust to altered sensory conditions. The cause of poor performance on surface sway-referencing is likely a reduced ability of subjects with PD to quickly re-weight toward increased reliance on vestibular cues at the start of each test. Poor performance on eyes-closed sway-referenced trials is also observed in older subjects without PD and with normal vestibular function (Peterka and Black, 1990).

### Re-weighting Sensitivity

Although subjects with PD were able to re-weight away from proprioception as surface-stimulus amplitude increased (Figures 6A,C), the difference in proprioceptive weights between the two smallest amplitudes of surface-stimuli was smaller in subjects with PD than age-matched controls but was the same for the two largest amplitudes (Figure 6B). This result is consistent with previous studies showing that subjects with PD have a higher threshold for perceiving the amplitude of proprioceptive stimuli (i.e., kinesthesia) than age-matched controls (Konczak et al., 2009), including an impaired ability to consciously perceive limb position (Maschke et al., 2003) or axial position (Wright et al., 2010). In addition, previous studies have demonstrated an increased neural synchrony in the basal ganglia circuitry with PD (Levy et al., 2000; Raz et al., 2001; Goldberg et al., 2004), suggesting a decrease in the signal-to-noise ratio of neural activity in the basal ganglia as dopamine levels decrease (Bergman et al., 1998; Bevan et al., 2002; Bar-Gad et al., 2003; Hammond et al., 2007). The higher threshold for detecting changes between the smallest proprioceptive stimuli that we observed in our study could be related to a decreased signal-to-noise ratio in the processing of sensory signals by dopaminergic circuitry.

### Stiffness in PD

The parameter K_p_, identified in model curve fits to FRF data (Figures 5, 7), characterizes the amount of corrective torque generated per unit of body sway and, thus, can be considered to quantify the stiffness of the postural control system (Latash and Zatsiorsky, 1993). That is, if one were to apply a perturbing torque of a given amplitude to subjects of equal body dimensions, a subject with a larger K_p_ would have a smaller sway amplitude than a subject with a smaller K_p_. Because rigidity is considered to be a hallmark of PD it may appear unexpected that K_p_ did not differ between subjects with PD and controls, and medication had no effect on K_p_ in subjects with PD.

However, in interpreting the meaning of stiffness and rigidity it is important to consider the context of the task that the subject is asked to perform, because the motor actions required for some tasks are highly constrained while motor actions for other tasks are not constrained. For example, when arm rigidity is tested, as in a UPDRS measurement of rigidity, control subjects allow free movement of their arms and low rigidity is observed, while subjects with PD resist movement of their arm, and high rigidity is observed. Similarly, tests show that subjects with PD off levodopa medication have increased axial rigidity when compared to controls, and levodopa does not significantly reduce axial rigidity (Wright et al., 2007). In both arm movement and trunk twisting tasks there are no functionally detrimental consequences if subjects allow arm or axial trunk movements in response to a perturbing force. That is, these tasks are not fundamentally constrained. Control subjects are apparently able to recognize the context of these situations and naturally allow free movement whereas subjects with PD are unable to adjust to the context.

In contrast, the task of maintaining upright stance places constraints on motor control. Both subjects with PD and controls must maintain a minimal K_p_ in order to resist the destabilizing torque due to gravity. Therefore, control subjects do not have an option of choosing a postural stiffness that is much less than that of subjects with PD. The upper limit of K_p_ is also constrained because large K_p_ values also produce instability (Masani et al., 2008). Therefore, only limited differences in K_p_ between subjects with PD and controls are possible. Furthermore, the very close correspondence between K_p_ values in subjects with PD and controls suggests that subjects with PD were able to regulate stiffness under steady-state conditions as well as control subjects to achieve dynamic control of upright stance (Peterka and Loughlin, 2004).

### Aging and Sensory Weighting

In response to a surface and visual stimuli, subjects with PD and age-matched controls show greater use of proprioceptive (Figure 6A) and visual (Figure 8) information (corresponding to larger W_prop_ and W_vis_ measures, respectively) compared to younger control subjects from a previous study (Peterka, 2002). For surface stimuli, greater sensitivity has been confirmed in more recent studies comparing younger and older adults with normal balance function (Cenciarini et al., 2010; Wiesmeier et al., 2015). These results indicate that subject age, not PD, determined the extent to which subjects utilized visual and proprioceptive information for postural control.

### Effects of Dopamine Replacement

Levodopa improved clinical indicators of balance and gait, as well as rigidity, bradykinesia and tremor, as measured by the UPDRS. However, consistent with the literature, we demonstrated that levodopa increases CoP displacement in the mediolateral direction during quiet stance, consistent with increased risk for falling (Mitchell et al., 1995; Rocchi et al., 2002). Previously, we showed that automatic postural responses to transient perturbations were further reduced by levodopa medication (Horak et al., 1996). Our current results indicate that levodopa neither improves nor impairs sensory weighting in PD patients in conditions where sufficient time is allotted to achieve steady-state behavior.

### Clinical Implications

Despite larger than normal postural sway during quiet stance and during larger sensory stimuli, our results show that subjects with PD do have the ability to change reliance on sensory information for postural control, given enough time to switch between tasks. This result does not conflict with our previous results showing that subjects with PD have difficulty switching quickly between different task demands (Horak et al., 1992; Chong et al., 1999b,c, 2000). Rather, it may be necessary for subjects with PD to transition slowly between tasks to avoid falls in the transition periods. In other words, postural instability in subjects with PD may be specific to the transition period. Consistent with this notion, we recently demonstrated that postural instability during walking in subjects with PD was specific to the transition period during heel-strike (Fino et al., 2018). If subjects with PD can ease through transition periods, they may be able to participate in activities that appear to challenge their postural stability (e.g., walking on a sandy beach). Conversely, if subjects with PD are required to produce a postural response during their transition period, they will likely demonstrate impaired postural responses that potentially increase the risk of falling. In addition, tasks requiring central processing of relatively small changes in sensory signals, such as walking from a firm to a more compliant surface, may be more affected by the loss of dopamine neurons, due to a decreased signal-to-noise ratio in the neural processing of sensory information and/or sensory integration signals. Consequently, patients may have trouble with tasks involving smaller changes in sensory conditions, but be successful at tasks in which there are larger changes in sensory conditions.

### Ethics Statement

The Institutional Review Board at Oregon Health and Science University (OHSU) approved the protocol for this experiment, and all subjects gave written informed consent prior to participating.

### Author Contributions

KF contributed to the analysis, interpretation of the results, and preparation of the manuscript. RP and FH both contributed to the research funding and resources, conception, experimental design, data collection, analysis and interpretation of the results, and editing of the manuscript.

## Conflict of Interest Statement

The authors declare that the research was conducted in the absence of any commercial or financial relationships that could be construed as a potential conflict of interest.

## Abbreviations

CoM: center of mass
CoP: center of pressure
FRF: frequency response function
J: body moment of inertia about ankle joints
K_d_: neural control damping constant
K_p_: neural control stiffness constant
K_T_: torque feedback gain
mgh: body mass times gravity constant times height of CoM above ankle joint
PD: Parkinson’s disease
PD_Off_: Parkinson’s disease subjects off medication
PD_On_: Parkinson’s disease subjects on medication
PIGD: postural instability and gait disorders
RMS: root-mean-square
τ_d_: neural control time delay
τ_T_: torque feedback time-constant
UPDRS: Unified Parkinson’s Disease Rating Scale
W_prop_: proprioceptive weight
W_vest_: vestibular weight
W_vis_: visual weight.
